# Impulsiveness and dysphoria as pharmacological targets: Can we aim at a phenomenologically informed therapeutic intervention?

**DOI:** 10.1192/j.eurpsy.2025.183

**Published:** 2025-08-26

**Authors:** S. Jerotic

**Affiliations:** 1 Faculty of Medicine, University of Belgrade; 2 Clinic for Psychiatry, University Clinical Centre of Serbia, Belgrade, Serbia

## Abstract

**Abstract:**

Dysphoria and impulsivity are embodied experiences that pervade numerous psychiatric conditions and evade easy categorization within traditional diagnostic boundaries. Phenomenological approaches are needed in order to clarify underlying experiential structures. Psychopharmacology has traditionally been perceived as a **“**biological intervention**”**, where success is measured by its impact on symptom clusters outlined in the ICD and DSM frameworks, ignoring the specific impact of medication on lived bodies and existential states. An embodied approach to psychopharmacology integrates phenomenology, neuroscience, and physiology, moving beyond traditional reductive perspectives. By examining how medications influence not only symptoms but also the lived experience and embodied sense of self, we can develop a more nuanced understanding of their effects, and possibly strive towards development of distinct phenomenological profiles of medication, which would enhance our understanding of dysphoria and impulsivity.

**Disclosure of Interest:**

None Declared

